# Cross-host transmission of *Riemerella anatipestifer* to chickens: Genomic evolution and identification of the novel *vapX*-like-*vapD* toxin-antitoxin system

**DOI:** 10.1080/21505594.2026.2711521

**Published:** 2026-07-30

**Authors:** Fanrun Meng, Ruiqi Li, Yancheng Yu, Xinyi Zhang, Wenjie Dai, Xiyao Cui, Lifeng Sun, Feng Lang, Liangyu Yang, Ziqiang Cheng

**Affiliations:** a College of Veterinary Medicine, Shandong Agriculture University, Tai’an, China; b College of Veterinary Medicine, Hebei Agricultural University, Baoding, China; c College of VeterinaryMedicine, Yunnan Agricultural University, Kunming, China

**Keywords:** *Riemerella anatipestifer*, cross-host transmission, antibiotic resistance genes, *VapD* gene, horizontal gene transfer

## Abstract

*Riemerella anatipestifer* (*R. anatipestifer*), a well-known waterfowl pathogen, increasingly threatens Chinese poultry by spreading to chickens. The genetic differentiation and host adaptation following cross-host transmission remain unclear. Here, we characterized a highly virulent, multidrug-resistant chicken-source strain (SDAU-RA1) and performed comparative genomics with global *R. anatipestifer* strains to elucidate population structure and evolutionary dynamics. SNP phylogeny revealed significant geographic clustering and dominant clonal groups. Strains from different hosts showed a pattern of “overall mixing with local clustering,” and ancestral state reconstruction (ASR) identified multiple independent duck-to-chicken spillover events, confirming cross-host transmission rather than strict host-specific evolution. In terms of virulence, certain virulence genes are enriched specifically in chicken-source strains. Notably, the study is the first to identify and confirm *vapX*-like-*vapD* as a functional type II toxin-antitoxin system in chicken-source *R. anatipestifer*, demonstrating that it enhances biofilm formation, intracellular survival, and antibiotic persistence. Analysis of the geographical distribution and temporal dynamics of antibiotic resistance genes (ARGs) reveals high heterogeneity among *R. anatipestifer* strains from different hosts. Pangenome analysis revealed that *R. anatipestifer* possesses an open pangenome, conferring high genetic plasticity. In conclusion, our study shows *R. anatipestifer* transmits to chickens without strict host-specific adaptation, though incipient genetic differentiation has emerged. The discovery of plasmid pRASD and its carried *vapX*-like-*vapD* system suggests key mechanisms for the adaptive evolution and enhanced pathogenicity of *R. anatipestifer*. These findings enhance our understanding of cross-host transmission and underscore the importance of continuous surveillance of chicken-source *R. anatipestifer* and its novel mobile genetic elements.

## Introduction

Pathogen host jumps driven by the selective pressures of intensive farming are intricately linked to the co-evolution of virulence and antimicrobial resistance, posing a formidable challenge to global public health [1]. *Riemerella anatipestifer* (*R. anatipestifer*), a Gram-negative bacterium, is traditionally recognized as a pathogen primarily affecting waterfowl, such as ducks and geese. Infections are typically characterized by fibrinous serositis and represent a significant threat to the global waterfowl industry [2]. However, recent epidemiological surveillance reveals that *R. anatipestifer* has breached its traditional host tropism, enabling cross-species transmission to chickens, a phylogenetically distant host [3]. An epidemiological investigation across 29 provinces in China from 2021 to 2024 demonstrated a significant expansion in the geographical distribution of *R. anatipestifer* from 10 to 19 provinces. The overall isolation rate surged from 1.03% to 4.56%, exhibiting a distinct seasonal pattern with peak incidence during the winter and spring months. Infections are increasingly observed in young, 3-to-6-week-old white-feathered broilers, where they primarily manifest as arthritis. In contrast, a high incidence of salpingitis (21.68%) is noted in layer breeders [4]. Furthermore, a detection rate of up to 20.18% in dead-in-shell embryos provides compelling evidence for vertical transmission [4]. The escalating infection rates and associated economic losses underscore the direct threat that the cross-host transmission of *R. anatipestifer* poses to the broiler and layer chicken industries in China.

The cross-host transmission of *R. anatipestifer* is directly linked to the co-evolution of virulence and antimicrobial resistance, a process driven by the pathogen’s genomic plasticity [5,6]. Research indicates that bacterial adaptation to new hosts is achieved through the modification of virulence genes. This process includes adaptive mutations in endogenous genes encoding key proteins, such as adhesins and outer membrane proteins, and the acquisition of novel virulence islands via HGT to enhance host affinity and immune evasion capabilities [7,8]. Concurrently, under the intense selective pressure of antibiotics in intensive farming, this adaptive process is synchronized with the evolution of resistance. Mobile genetic elements (MGEs), such as plasmids, prophages, and insertion sequences, frequently co-locate ARGs and virulence factors (VFs), establishing genetic structures with “virulence-resistance co-linearity.” This genetic linkage drives the evolution of pathogens toward phenotypes of high virulence and high resistance [9,10]. Notably, the discovery that *tet(X)* family genes, which confer resistance to last-resort antibiotics like tigecycline, are integrated into these MGEs underscores the central role of the mobile genome in shaping high-risk pathogens [11]. Importantly, a recent systematic analysis of the *R. anatipestifer* resistome landscape in China revealed a remarkably high prevalence of *bla*_*OXA*_-like (93.1%) and *tet(X)* (90.6%) genes [12]. However, there has been limited research comparing the resistomes of *R. anatipestifer* isolates from different hosts, particularly between its traditional waterfowl hosts (ducks and geese) and its emergent host, the chicken.

Currently, despite the rising prevalence of *R. anatipestifer* in chicken populations, the molecular mechanisms driving its cross-host transmission are not well-defined. There is a notable absence of systematic comparative genomic studies analyzing strains from its diverse avian hosts, namely chickens, ducks, and geese. To achieve this, we first isolated and identified a chicken-source strain, SDAU-RA1, from a clinical case and subsequently validated its pathogenicity through an animal challenge study. Building on this foundation, we sequenced its complete genome. This enabled us to perform the first comprehensive, genome-level comparative analysis of strains from these distinct hosts, integrating our data with a large collection of publicly available duck- and goose-source genomes. The overarching objective was to uncover the key genetic determinants that shape the host range and pathogenic potential of *R. anatipestifer*.

## Materials and methods

### Isolation, characterization, and pathogenicity assessment of the chicken-source *R. anatipestifer* strain SDAU-RA1

#### Isolation and identification

Bacterial isolation and identification were conducted using samples collected from a broiler farm in Tai’an City, Shandong Province, China, during an outbreak of *R. anatipestifer*. Diseased chickens exhibited typical symptoms of systemic infection, with necropsy findings revealing fibrinous pericarditis and arthritis. Under aseptic conditions, exudates from the liver surface were collected and inoculated onto Tryptic Soy Agar (TSA) plates supplemented with 5% defibrinated sheep blood. The plates were incubated at 37°C in a 5% CO_2_ atmosphere for 24–48 hours. Typical small, grayish-white colonies with smooth, moist surfaces and regular edges were selected and subcultured to obtain a pure culture. Genomic DNA was extracted using a Bacterial Genomic DNA Extraction Kit (TIANGEN Biotech, Beijing, China) according to the manufacturer’s instructions. PCR amplification was performed using 16S rRNA primers (Forward: 5′-AGAGTTTGATCCTGGCTCAG-3′; Reverse: 5′-GGTTACCTTGTTACGACTT-3′) [13].

#### Antimicrobial susceptibility testing

The antimicrobial susceptibility of the isolate was evaluated using the Kirby-Bauer disk diffusion method. In accordance with the Clinical and Laboratory Standards Institute (CLSI) guidelines (2023), the susceptibility of isolate SDAU-RA1 to 21 antibiotics, representing 10 different classes, was determined to establish its phenotypic resistance profile. *Escherichia coli* (*E. coli*) ATCC 25922 was used as the quality control strain.

#### Pathogenicity test

To evaluate the pathogenicity of isolate SDAU-RA1, an animal infection model was established using 1-day-old specific-pathogen-free (SPF) chicks. Animals were obtained from Shandong Minhe Animal Husbandry Co., Ltd. (Shandong, China) (average body weight: 40 g; sex: mixed). The SPF chicks were randomly divided into two groups. Chicks in the infection group (*n* = 18) were intramuscularly injected with 0.1 mL of the SDAU-RA1 bacterial suspension at a concentration of 1 × 10^6^ CFU/mL [3]. The control group (*n* = 18) received an equal volume of sterile phosphate-buffered saline (PBS). Following inoculation, all chicks were monitored daily for clinical signs and general condition for a period of 45 days. Moribund or dead birds were necropsied to examine for typical pathological lesions. All experimental chickens were housed in a three-dimensional caging system. To control for potential cage-level effects, cages from all groups were interspersed on the racks. All procedures were conducted in strict accordance with the guidelines of the Animal Care and Welfare Committee of Shandong Agricultural University. The infected group was compared to the control group based on multiple indicators, including clinical signs (such as appetite and general health status) and pathogen re-isolation. In accordance with the American Veterinary Medical Association (AVMA) Guidelines (Section S3.4), chickens exhibiting clinical signs such as depression, lameness, or neurological symptoms were humanely euthanized by cervical dislocation and subsequently necropsied [14]. During necropsy, bacteria were isolated and identified, and tissue samples from the heart, liver, joints, brain, and spleen were collected for histopathological analysis.

### Whole genome sequencing and analysis

Genomic DNA of SDAU-RA1 was extracted using a modified CTAB method. The quality and quantity of the DNA were assessed using a Qubit fluorometer (Invitrogen, USA) and a NanoDrop spectrophotometer (Thermo Scientific, USA). Hybrid second- and third-generation sequencing was performed on the Illumina NovaSeq and Nanopore PromethION platforms. Following quality control of raw data with AdapterRemoval and SOAPec, a hybrid assembly strategy was employed. A draft genome was constructed by assembling the third-generation long reads using Flye and Unicycler. The resulting assembly was then polished and corrected using the second-generation short reads with Pilon software to obtain a high-quality, complete genome. As of June 2025, a total of 443 complete genome sequences of *R. anatipestifer* were downloaded from the public NCBI Pathogen Detection database. The genomes were screened using CheckM v.1.2.3 (https://github.com/Ecogenomics/CheckM) based on quality criteria of ≥95% completeness and ≤5% contamination. This resulted in a final dataset of 283 high-quality genomes selected for subsequent analysis, comprising isolates from chickens (*n* = 9), ducks (*n* = 238), and geese (*n* = 36). Detailed information for all *R. anatipestifer* genomes used in this study, including strain isolation date, country of origin, and host, is provided in Supplementary Table 1. We plotted the global geographic distribution of the isolates using the maps and ggplot2 packages in R, and generated a Sankey diagram illustrating their source distribution using the ChiPlot online tool (https://www.chiplot.online/).

### Genome annotation and characterization

The genome of isolate SDAU-RA1 (GenBank: CM126355.1) from this study (Supplementary Figure 1C), along with the 280 downloaded genomes (after removing three strains with identical names), was annotated using Prokka v1.14.6 [15]. Multi-Locus Sequence Typing (MLST): Sequence types (STs) were assigned to all 280 genomes using MLST v2.0 (https://github.com/tseemann/mlst) based on the seven housekeeping genes defined in the *R. anatipestifer* PubMLST database (https://pubmlst.org/ranatipestifer/). Virulence and Resistance Gene Prediction: VFs and ARGs were predicted via local BLASTp searches against the Virulence Factor Database (VFDB) and the Comprehensive Antibiotic Resistance Database (CARD) [16,17], respectively. Hits for VFs were filtered using thresholds of ≥60% identity and ≥70% coverage. For ARGs, the criteria were ≥45% identity and ≥70% coverage. For both analyses, an E-value cutoff of <1 × 10^−5^ was applied, and only the best-scoring hit for each query was retained. MGE Identification: To identify MGEs, plasmid replicons were detected using PlasmidFinder v2.1 against the PlasmidFinder database [18]. Prophage regions (intact, incomplete, and questionable) were predicted using the PHASTER (Phage Search Tool Enhanced Release) web server (https://phaster.ca/). Insertion sequences (ISs) were identified using ISEScan v1.7.2.3 in conjunction with the ISfinder database [19].

### Phylogenetic and evolutionary analysis

To elucidate the evolutionary history and transmission pathways of 280 *R. anatipestifer* isolates, we constructed a high-resolution phylogenetic tree based on core genome single nucleotide polymorphisms (SNPs). High-quality sequencing reads from 279 genomes were mapped to the complete genome of *R. anatipestifer* DSM_15868 (used as the reference) with Snippy (v4.6.0) for variant calling [20]. The snippy-core command was then used to generate a robust multiple sequence alignment from the core SNPs conserved across all isolates. Using this alignment, a Maximum Likelihood (ML) phylogenetic tree was reconstructed with FastTreeMP (v2.1.11) (https://github.com/gtonkinhill/fastbaps). The tree was inferred using the General Time Reversible (GTR) model of nucleotide substitution, coupled with a GAMMA model to account for rate heterogeneity across sites. Branch support was assessed using 1000 ultrafast bootstrap replicates. Finally, the resulting Newick-formatted tree was visualized and annotated using the Interactive Tree of Life (iTOL) v6 (https://itol.embl.de/).

### Ancestral state reconstruction

To infer the directionality and frequency of cross-host transmission events, ancestral state reconstruction (ASR) of the host character was performed using the PastML tool (v1.9.30) (https://pastml.pasteur.fr/). The maximum-likelihood phylogenetic tree and host metadata (duck, goose, and chicken) were utilized as inputs. The reconstruction was conducted using the marginal posterior probabilities approximation (MPPA) method under the F81 decision model.

### Comparative genomics and genetic context analysis

A clustering heatmap was generated to visualize the distribution of ARGs and VFs across the 280 *R. anatipestifer* isolates (Supplementary Table 2). The hierarchical clustering of the isolates (left-side dendrogram) was performed based on the presence/absence profiles of the ARGs and VFs. A binary matrix was constructed (“1” for presence, “0” for absence), and a dissimilarity matrix was calculated using the Jaccard distance. Finally, clustering was performed using the complete linkage method. Additionally, the prevalence of each ARG and VF (defined as the percentage of isolates harboring the gene) was calculated (Supplementary Tables S3–S6), and a prevalence heatmap was generated using the ChiPlot online tool. To elucidate the genetic context of the tigecycline-resistance gene *tet(X)*, the flanking sequences from six *R. anatipestifer* isolates of different host origins—comprising two from ducks, three from chickens (including SDAU-RA1, isolated in this study), and one from a goose—were subjected to comparative and synteny analysis using Easyfig v2.2.5 [21]. A circular genomic comparison map was generated using BRIG (BLAST Ring Image Generator) v0.95 (https://sourceforge.net/projects/brig/) to visualize the comparative genomics of isolates from different sources (chickens, *n* = 3; ducks, *n* = 2; and geese, *n* = 2) against a reference genome. The map was annotated to highlight the genomic locations of predicted virulence factors and MGEs on the SDAU-RA1 genome. Furthermore, a linear comparison of ten representative plasmids (Supplementary Table 7), including the novel plasmid pRASD identified in this study, was performed to analyze their genetic organization and functional modules.

### Functional identification and biological characterization of the *vapX-like/vapD*toxin-antitoxin system

#### Structural modeling of the *vapX-like/vapD* complex

The *vapD* toxin gene was discovered in the virulence determinant area of pRASD. Just upstream of *vapD* is an open reading frame, according to genomic studies. The amino acid sequence contained within the encoding shows low levels of homology to any known *vapX* antitoxins; however, the genetic organization is a typical feature of Type II toxin-antitoxin (TA) systems. To confirm structurally whether this upstream gene product (referred to as *vapX*-like) interacts with *vapD* to form a functional complex, a 3D model of the *vapX*-like/*vapD* heterodimer was built using AlphaFold 3 [22]. Then, the PyMOL molecular visualization platform was used to systematically characterize critical interactions at the binding interface based on stringent geometric parameters. The analysis clarified the intermolecular forces that are fundamental for the stability of complexes. These include hydrogen bonds, salt bridges, and π-π stacking.

#### Heterologous functional verification of the toxin-antitoxin system

To determine whether *vapX*-like and *vapD* constitute a functional TA system, an *E. coli*-based dual-plasmid co-expression system was constructed. The *vapD* gene was inserted in a pBAD33 vector (Cm^R^, araBAD promoter) to construct a toxin expression plasmid pBAD33-*vapD* while *vapX*-like gene was inserted in a pKK223-3 vector (Amp^R^, tac promoter) to construct an antitoxin expression plasmid pKK223-3-*vapX-like*. All primer sequences and relevant information are listed in Supplementary Table 8. These plasmids and their corresponding empty vector controls were co-transformed into *E. coli* BW25113. To ensure stable plasmid maintenance and repress basal toxin expression, all cultures were supplemented with chloramphenicol (34 μg/mL), ampicillin (100 μg/mL), and 0.2% D-glucose. Cell growth and viability were assessed using spot dilution assays and liquid induction-CFU counting methods. Overnight cultures were washed with PBS to remove glucose, normalized to an OD_600_ of 1.0, and serially diluted 10-fold. Aliquots (20 μL) were spotted onto LB agar plates containing specific inducers (0.2% L-arabinose for toxin, 1 mM IPTG for antitoxin, or both) and incubated at 37°C for 16 hours. For viability assays, cultures were grown to early log phase and induced for 2 hours. Samples were diluted and plated onto double-antibiotic plates containing 1% glucose (to terminate toxin expression), and CFUs were enumerated to calculate survival rates.

#### Construction of the vapX-like-vapD deletion mutant in *R. anatipestifer*

A scarless *vapX*-like-*vapD* deletion mutant was constructed through homologous recombination using the suicide plasmid pRE112. Using pRASD as a template, primers were used to amplify the upstream homology arm of *vapX*-like (L-arm, with *SacI* site) and the downstream homology arm of *vapD* (R-arm, with *XbaI* site). Splicing by overlap extension PCR (SOE-PCR) was used to fuse the upstream and downstream fragments. The fused product was then digested and ligated into pRE112 to create the recombinant suicide plasmid pRE112-Δ*vapX*-like-*vapD*, which was subsequently transferred into *E. coli* S17-1 λpir. For conjugation and selection, donor cells (*E. coli* S17-1) and recipient cells (*R. anatipestifer* SDAU-RA1) were mixed at a 1:10 ratio and co-cultured on antibiotic-free TSA serum plates for 14 hours. Single-crossover transconjugants were selected on TSA serum plates containing kanamycin (50 μg/mL) and chloramphenicol (15 μg/mL). Positive clones were passaged six times in antibiotic-free medium and then plated onto medium containing 8% sucrose (without NaCl) for *sacB*-mediated counter-selection. The markerless deletion mutant Δ*vapX*-like-*vapD* was confirmed by PCR and sequencing.

#### Construction of the complementation strain

To verify the phenotype, a complementation strain was constructed using the shuttle vector pCP29. The full-length *vapX*-like-*vapD* fragment, including its native promoter, was amplified using primers C-*vapX*-like-Pro380-F (*KpnI*) and C-*vapD*-R (*SphI*). The fragment was digested and ligated into pCP29, transformed into *E. coli* DH5α, and selected with cefoxitin (1 μg/mL). The verified plasmid was transformed into the donor strain *E. coli* S17-1λpir and conjugated with the deletion mutant. Bacterial lawns were eluted and plated onto TSA plates containing 1 μg/mL cefoxitin and 40 μg/mL polymyxin B. Polymyxin B was used to specifically eliminate the *E. coli* donor (to which *R. anatipestifer* is naturally resistant), thereby isolating the pure complementation strain C-*vapX*-like-*vapD*.

#### Genetic and physiological characterization of the strains

To determine the relative copy number of the pRASD plasmid, the wild-type (WT) strain SDAU-RA1, the Δ*vapX*-like-*vapD* mutant, and the complemented C-*vapX*-like-*vapD* strain were cultured in TSB to the mid-logarithmic phase. Genomic DNA was extracted using the TIANamp Bacteria DNA Kit (TIANGEN, China). qPCR was performed targeting the plasmid-specific *nisB* gene and the chromosomal single-copy *gyrB* gene. The relative plasmid copy number per chromosome was calculated as 2^Δ*Ct*^, where Δ*C*_*t*_=*C*_*t*_(*gyrB*)−*C*_*t*_ (*nisB*). All primers used in these assays are listed in Supplementary Table 8. For the plasmid stability assay, the strains were cultured in antibiotic-free TSB and passaged via 1:1,000 dilution every 24 h for 3 consecutive days (corresponding to approximately 30 generations). The final cultures were plated on TSA, and 24 colonies per strain were randomly selected and screened by colony PCR targeting *nisB* to determine the plasmid retention rate. To assess potential polar effects, the strains were grown to the mid-logarithmic phase. Total RNA was extracted using the RNAiso Plus reagent (TaKaRa, Japan) and reverse-transcribed into cDNA using the PrimeScript RT Reagent Kit with gDNA Eraser. RT-qPCR was performed to analyze the transcript levels of the genes (*apxIB* and *hin*) flanking the *vapX*-like-*vapD* locus, using *gyrB* as the internal reference, with relative expression levels calculated using the 2^−ΔΔ*Ct*^ method. To evaluate growth fitness, overnight cultures of the strains were diluted 1:100 in fresh TSB and incubated at 37°C with shaking at 180 rpm. The OD_600_ was monitored every 2 h over a 24-h period.

#### Biofilm formation assay

Biofilm formation capability was evaluated using a crystal violet staining assay. Overnight cultures of WT, mutant, and complementation strains were normalized to OD_600_ of 0.1 in fresh TSB and dispensed into the rows of 96-well polystyrene plates (200 μL/well). The plates were incubated for 24 hours at 37°C. The supernatant was removed carefully, and the wells were washed gently with PBS three times to remove the planktonic bacteria. Then the wells were fixed with methanol for 15 minutes. The biofilms were stained with 0.1% crystal violet for 15 minutes and washed with water. The dye bound was made soluble with 33% acetic acid. The absorbance was measured at 570 nm (OD_570_). Four biological replicates were used for experiments.

#### Intracellular survival, persister, and oxidative stress assays

Intracellular survival in chicken macrophage HD11 cells was evaluated using a cefazolin protection assay. HD11 monolayers were infected with log-phase cultures of the WT, mutant, and complementation strains at an MOI of 100:1 and incubated at 37°C for 1 hour to allow phagocytosis. After washing with PBS, cells were incubated with medium containing 200 μg/mL cefazolin for 2 hours to completely kill extracellular bacteria (defined as T0), followed by incubation in maintenance medium containing 20 μg/mL cefazolin. At designated time points, cells were lysed with 0.1% Triton X-100, and lysates were serially diluted and plated onto antibiotic-free TSB agar to determine intracellular bacterial loads. For the oxidative stress assay, log-phase cultures (OD_600_ ≈ 0.8) were treated with 2 mM H_2_O_2_ at 37°C for 1 h. The survival rate was calculated as the percentage of CFUs in the treated group relative to the untreated control. For the time-kill assay, log-phase cultures of each strain were exposed to 100 μg/mL cefazolin to simulate antibiotic stress. Samples were collected at 0, 2, 6, 12, and 24 hours, washed with PBS to remove antibiotics, and plated for enumeration to generate time-kill curves assessing persister formation.

#### In vivo colonization assay

To evaluate the role of the *vapX*-like-*vapD* system in systemic colonization, a chicken infection model was established according to the pathogenicity test protocol described in section: “Isolation, characterization, and pathogenicity assessment of the chicken-source R. anatipestifer strain SDAU-RA1”. Briefly, twelve 7-day-old healthy chickens were randomly divided into four groups (*n* = 3 per group) and intramuscularly inoculated with 2 × 10^8^ CFU of the WT strain, the mutant strain, the complemented strain, or sterile PBS (as a negative control), respectively. At 48 hpi, all chickens were euthanized. Heart blood, liver, and spleen samples were aseptically collected. Tissues were homogenized in 1 mL of sterile PBS. The blood samples and tissue homogenates were serially diluted and plated on TSA plates to determine bacterial counts (CFU/mL). Animal experiments were approved by the Institutional Animal Care and Use Committee.

### Pangenome analysis

To construct a representative *R. anatipestifer* genomic dataset while minimizing analytical redundancy from sampling bias and maximizing genetic diversity, we integrated strain origin information with Average Nucleotide Identity (ANI) values. This process yielded a non-redundant dataset comprising 130 high-quality genomes for downstream analysis. The dataset represents diverse geographical locations and host origins, including 8 strains from chickens, 105 from ducks, and 17 from geese. A pan-genome analysis was performed on this dataset using Roary (v3.13.0) with a BLASTp identity threshold of 95% [23]. Genes were categorized as core (present in ≥99% of strains), shell (15–99%), or cloud (<15%), generating a gene presence/absence matrix and a core gene alignment. Based on the core gene alignment, a Maximum Likelihood (ML) phylogenetic tree was constructed using IQ-TREE (v2.2.0). The best-fit substitution model was determined by ModelFinder, and branch support was assessed with 1000 ultrafast bootstrap replicates. Furthermore, to evaluate the openness of the species’ pan-genome, an accumulation curve was plotted using the output data from Roary.

#### Statistical analysis

All experiments were performed in three independent replicates. The final morbidity rates between groups were compared using Fisher’s exact test. For histopathological analysis, tissue samples were collected from representative chickens in each group, and representative images are presented. Statistical analyses were conducted using GraphPad Prism 8.0 (GraphPad Software, USA). Data are presented as mean ± SD. One-way ANOVA followed by Tukey’s multiple comparisons test was used to analyze differences among multiple groups at a single time point. Two-way ANOVA with Tukey’s multiple comparisons test was used to analyze data involving multiple groups and time points. A *p*-value < 0.05 was considered statistically significant (*, *p* < 0.05, **, *p* < 0.01, ***, *p* < 0.001, ****, *p* < 0.0001; ns, not significant).

## Results

### Biological characterization of the chicken-source *R. anatipestifer* isolate SDAU-RA1

Phylogenetic analysis based on the 16S rRNA gene sequence showed that the isolate was most closely related to the *R. anatipestifer* reference strain (CP011859), clustering into a distinct branch with a high confidence value of 0.91 (Supplementary Figure 1(A)). Based on these findings, it was identified as *R. anatipestifer*. Antimicrobial susceptibility testing revealed a severe resistance profile, with the isolate exhibiting a typical multidrug-resistant (MDR) phenotype. Its resistance spectrum was extremely broad, demonstrating resistance to at least six major classes of antibiotics, including aminoglycosides, macrolides, tetracyclines, β-lactams, fluoroquinolones, and glycopeptides (Supplementary Figure 1(B)). Notably, the isolate remained susceptible only to a few drugs, including lincosamides (e.g. clindamycin), oxazolidinones (linezolid), and sulfonamides (sulfathiazole).

**Figure 1.**
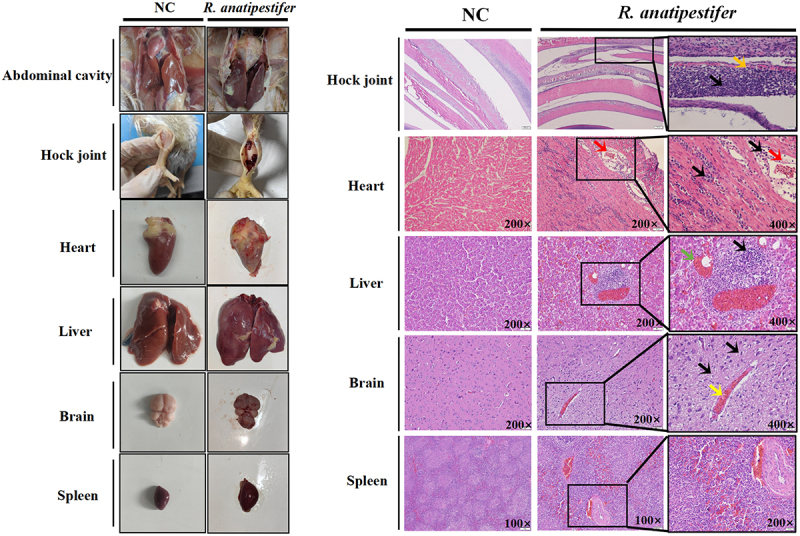
Histopathological lesions in chicks infected with SDAU-RA1.

To evaluate the pathogenicity of isolate SDAU-RA1, 1-day-old SPF chicks were infected via intramuscular injection. Chicks in the infected group (10^6^ CFU/bird) exhibited clinical signs of depression and ruffled feathers on day 8 post-infection, followed by lameness on day 17. The final morbidity rate of the infected group (100%, 18/18) was significantly higher than that of the control group (0/18) (*p* < 0.0001, Fisher’s exact test). No mortality occurred in either group during the 45-day observation period. Gross necropsy revealed pericarditis, fibrinous perihepatitis, swollen hock joints containing blood (hemarthrosis), and cerebral hemorrhage (Figure 1). Histopathological examination of the joints revealed proliferation of fibrous connective tissue (orange arrow), accompanied by extensive inflammatory cell infiltration (black arrow). The heart showed perivascular edema (red arrow), with extensive inflammatory cell infiltration in the interstitial spaces between myocardial fibers (black arrow). In the liver, hepatic cord disarray and congestion in the sinusoids and blood vessels were visible (green arrow); focal perivascular inflammatory cell infiltration was also noted (black arrow). The brain exhibited numerous shrunken and hyperchromatic neurons (black arrow), which were irregular in shape and reduced in size. Vascular congestion was also observed (yellow arrow). In the spleen, the distinction between the red and white pulp was blurred, and the vascular walls were thickened with peripheral hemorrhage.

Gross pathology of affected organs is shown on the left, with corresponding histopathological examinations on the right.

### Population structure and evolutionary relationships of *R. anatipestifer*

**Figure 2.**
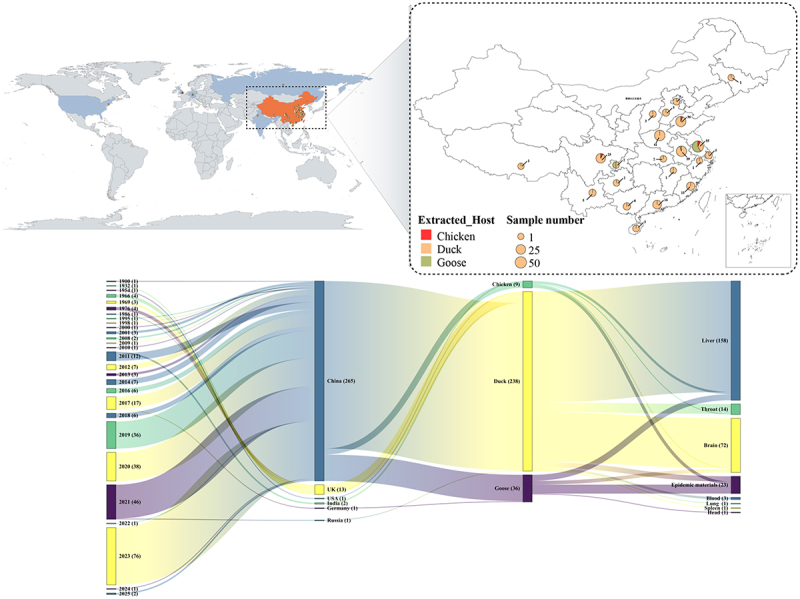
Epidemiological characteristics of *R. anatipestifer.*

**Figure 3.**
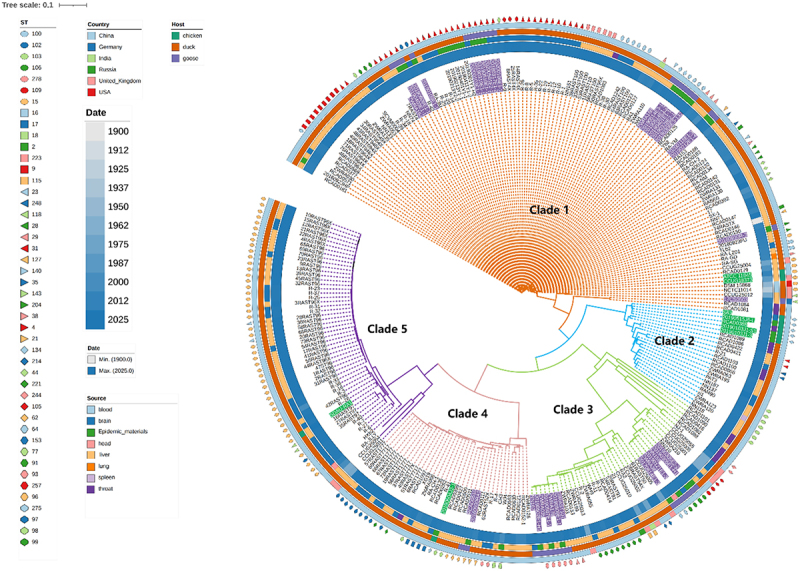
SNP-based phylogenetic tree of *R. anatipestifer* isolates from different hosts.

To systematically analyze the population characteristics of *R. anatipestifer*, we investigated 283 isolates, revealing a highly concentrated epidemiological distribution. Geographically, the vast majority of isolates (265 strains, 93.6%) originated from China, with only sporadic distribution from other countries (Figure 2). Temporally, although the earliest strain dates back to 1900, the vast majority (approx. 83%) were collected after 2017, with a surge in recent years. Regarding host origin, ducks were the primary host (84.1%), followed by geese. Although only 9 chicken-source isolates are in public databases, field surveys show that over 700 were identified between 2021–2024 [4]. This indicates a significantly increasing risk of cross-host transmission from chicken-source strains—a threat not fully reflected by current genomic data. MLST analysis of 280 isolates in this study identified 95 STs (Supplementary Table 9), with ST96 (15.7%) and ST4 (11.7%) being the most prevalent. ST4, ST64, and ST278 were the primary types in goose isolates, while ST134 was the main type in chicken isolates. A phylogenetic tree based on core genome SNP partitioned all isolates into five major evolutionary clades, showing strong concordance with MLST results, as isolates of the same ST typically formed distinct sub-clades (Figure 3). For instance, Clade 1 comprised multiple STs, whereas Clade 5 consisted almost entirely of ST96, suggesting it is a highly successful and conserved lineage.

The map shows the geographical distribution of the *R. anatipestifer* isolates analyzed. A Sankey diagram illustrating the associations among the collection year, country of origin, host species, and tissue source for the isolates.

The isolates are partitioned into five major evolutionary clades (Clade 1–5), which are distinctly color-coded. The tree is annotated with concentric rings that display metadata for each isolate. From the innermost ring outward, these represent: isolation date, tissue of origin, host (orange: chicken; blue: duck; purple: goose), and country of origin. ST of each isolate is indicated by distinct shapes on the outermost ring.

From a host distribution perspective, isolates did not form strictly demarcated clusters. Duck isolates were found across all five clades, highlighting the duck’s role as a primary host, with chicken and goose isolates interspersed among them. However, despite this general admixture, clear host-specific clustering was observable. Specifically, goose isolates concentrated in Clades 1 and 3, forming waterfowl-specific branches with duck isolates, and chicken isolates also showed clustering tendencies. Further analysis revealed that these clustered goose and chicken isolates predominantly originated from Jiangsu Province, China (Supplementary Figure S2), indicating that geographical factors significantly influence their evolution and aggregation. This pattern of “overall admixture and local clustering” provides strong evidence for extensive cross-host transmission of *R. anatipestifer*, rather than strict host-adaptive evolution.

### ASR of *R. anatipestifer* transmission

ASR revealed a clear transmission network of *R. anatipestifer* across different hosts (Supplementary Figure S3). A large ancestral node dominated by duck isolates (duck 204) was identified at the core of the network, indicating that ducks serve as the primary reservoir. Crucially, multiple independent transition events from ducks to chickens were observed, including a major transmission pathway to the “chicken 1–5” cluster (weight = 3) and another to the “chicken 2” node (weight, representing the number of inferred transition events). Additionally, frequent transmission events were detected between ducks and geese, such as transitions from the “duck 204” node to “goose 1–7” (weight = 5) and from “goose 8” back to duck populations. These findings provide direct evolutionary evidence for *R. anatipestifer* spillover from waterfowl to chickens.

### Geographic distribution and temporal dynamics of ARGs

**Figure 4.**
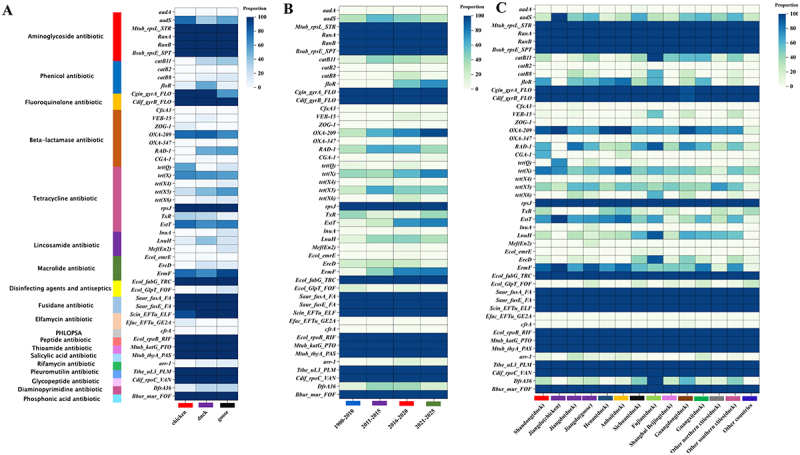
Spatiotemporal and host-specific patterns of ARGs in *R. anatipestifer.*

We systematically analyzed the resistome and virulome of 280 *R. anatipestifer* genomes. A clustering heatmap revealed that ARGs and VFs have highly heterogeneous distributions. Most chicken-source isolates with similar profiles clustered closely on the phylogenetic tree, forming adjacent branches (Supplementary Figure S4), a trend also observed for goose-source isolates. Notably, both of these host-associated clusters fall within the same major evolutionary clade, indicating they remain closely related despite undergoing host-specific adaptation. Resistome analysis identified 18 core ARGs, covering resistance to aminoglycosides (*rpsL*, *RanA*, etc.) and fluoroquinolones (*gyrA*, *gyrB*) (Figure 4(A)), with minimal overall differences among isolates from different hosts. A longitudinal analysis (Figure 4(B)) showed that while the 18 core genes persisted, the prevalence of other key ARGs changed dramatically. The carriage rate of the florfenicol resistance gene *floR* surged to over 65% in the 2021–2025 period, while the β-lactamase gene *bla*_*OXA-209*_ has shown a continuous linear increase, now exceeding 80%. Similarly, genes such as *tet(X)*, *EstT*, and *ErmF* also displayed significant upward trends. These dynamic changes indicate that antimicrobial selection pressure over the past decade is reshaping the bacterium’s resistome.

Heatmaps showing the prevalence of ARGs in *R. anatipestifer*. Genes (y-axis) are grouped by antibiotic class. Color scale represents the prevalence rate (%), with darker colors indicating higher rates. (A) ARG prevalence in 280 isolates from different hosts (chicken, duck, goose). (B) ARG prevalence across four time periods. (C) ARG prevalence in isolates from different geographical regions.

The distribution of ARGs exhibited significant geographic heterogeneity. Isolates from provinces like Jiangsu, Anhui, and Fujian had significantly higher carriage rates of genes conferring resistance to β-lactams, chloramphenicols, tetracyclines, and macrolides (Figure 4(C)). Among these, chicken-source isolates from Jiangsu showed carriage rates approaching 100% for *aadS*, *bla*_*OXA-209*_, *EstT*, and *ErmF*, reflecting extremely strong selection pressure. The Fujian region was characterized by high carriage rates of genes like *catB*, *bla*_*RAD-1*_, and *lnu(H)*. Finally, compared to isolates from other countries, Chinese strains carried a significantly higher diversity and prevalence of ARGs, highlighting the immense antibiotic selection pressure within China’s farming industries.

### Genetic context of the tet(X) gene in *R. anatipestifer*

**Figure 5.**
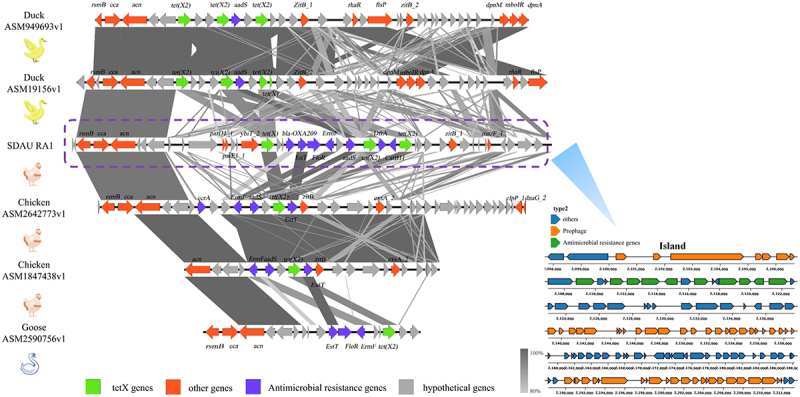
Genetic context of the *tet(X)* gene in *R. anatipestifer* isolates from different hosts.

To investigate the genetic context of the *tet(X)* gene in *R. anatipestifer* from different hosts, we analyzed its flanking regions (Figure 5). The analysis revealed that *tet(X)* and its associated resistance gene cluster are located downstream of a conserved *rsmB-cca-acn* backbone sequence. This suggests that *tet(X)* preferentially integrates into specific genomic hotspots, often co-localizing with other ARGs like *aadS* or *ErmF* to form diverse resistance modules. In duck-source isolates, *tet(X)* was typically the *tet(X2)* variant, and its downstream region was also relatively conserved.

Genes are color-coded according to function (see key), and the shaded gray areas between isolates represent sequence homology. A unique GI flanked by prophage elements was identified in the chicken isolate SDAU-RA1. This GI, magnified in the bottom right inset, carries a total of ten distinct ARGs, including *tet(X)*, bla_OXA-209_, *ErmF*, and *DfrA*, forming an extensive composite resistance region. Upstream of this region, the plasmid partitioning-related genes *parD1* and *parE1* are also present.

Notably, the genetic environment of *tet(X)* in the chicken-source isolate SDAU-RA1 was highly distinct. Its *tet(X)* gene cluster was located within a GI flanked by prophage elements, forming a vast composite resistance region far exceeding other isolates in size and complexity. In addition to *tet(X)*, this GI was densely packed with multiple resistance genes, including *bla*_*OXA-209*_, *ErmF*, *DfrA*, another copy of *tet(X2)*, and *catB11*. Furthermore, genes related to a plasmid partitioning system, *parD1*/*parE1*, were located upstream, which is a structural combination not found in the other isolates.

### Virulome diversity of *R. anatipestifer* isolates from different hosts and characterization of the virulence plasmid pRASD in isolate SDAU-RA1

**Figure 6.**
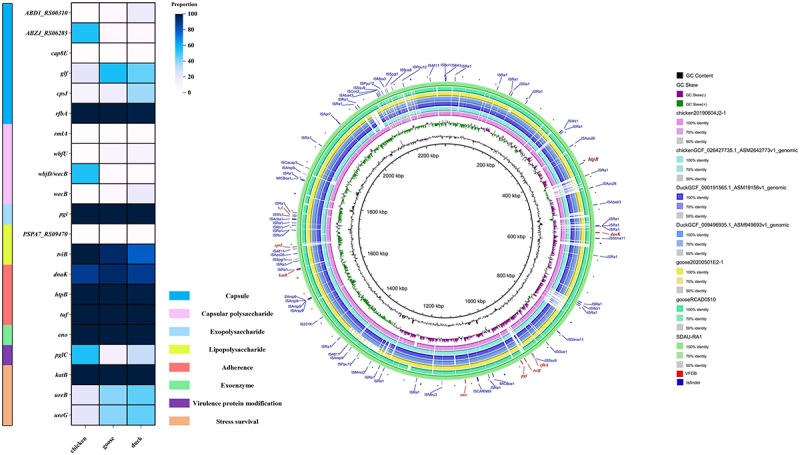
Analysis of differential virulence gene profiles in *R. anatipestifer* and the genetic context of strain SDAU-RA-1.

**Figure 7.**
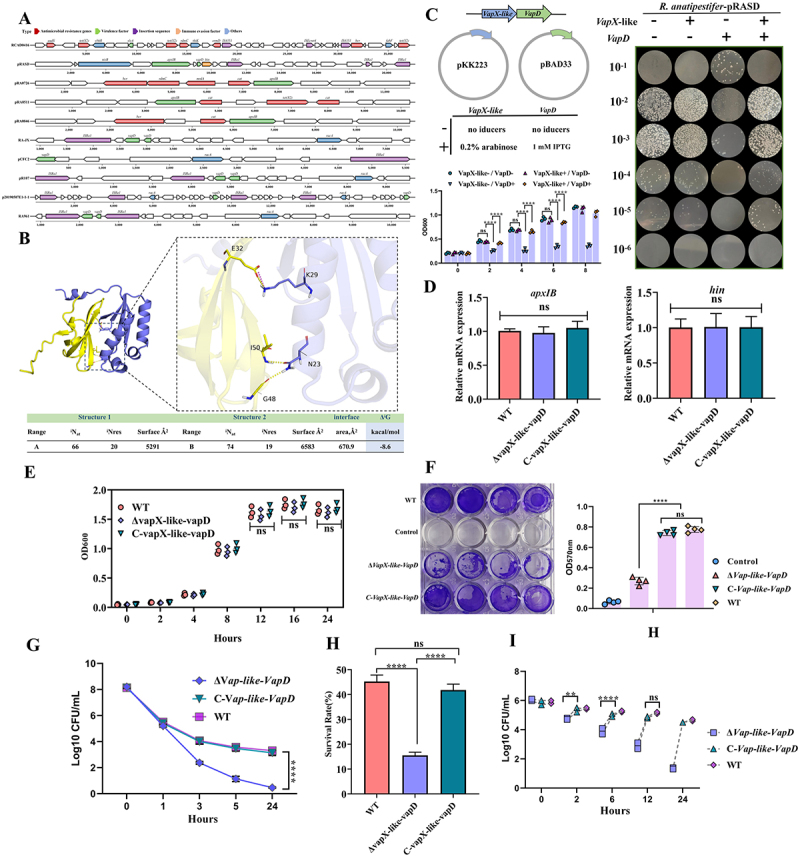
Genetic characterization of the virulence plasmid pRASD and functional analysis of the *vapX*-like-*vapD* toxin-antitoxin system.

To investigate host adaptation, we analyzed the virulence gene profiles of strains from different origins. Heatmap analysis revealed that chicken-source isolates, as a cohort, were significantly enriched in three key virulence genes compared to waterfowl (duck and goose) isolates: ABZJ_RS06285 (*TssL* protein), *wbjD/wecB* (capsular polysaccharide synthesis enzyme), and *pglC* (glycosyltransferase) (Figure 6). In contrast, the virulence profiles of goose- and duck-source strains were highly similar. Crucially, however, the chicken-source strain isolated in this study, SDAU-RA1, does not harbor these three prevalent genes. This indicates that the pathogenic strategies of *R. anatipestifer* in chickens are diverse and do not rely on a single evolutionary pathway. Further genomic comparison revealed that other virulence genes in SDAU-RA1 are located in proximity to MGEs such as IS, which strongly supports the hypothesis that HGT is a key driver for its acquisition of virulence and adaptation to new hosts. More importantly, we discovered that our isolate, SDAU-RA1, harbors a plasmid, which we designated pRASD. This represents the first report of a plasmid in a chicken-source *R. anatipestifer* strain. To elucidate the genetic characteristics of *R. anatipestifer* plasmids, we therefore conducted a linear genomic comparison of 10 representative plasmids (Figure 7(A)). This analysis revealed that the plasmids can be broadly categorized, based on their structure and function, into two main types: virulence-associated and antimicrobial resistance plasmids, with some exhibiting hybrid characteristics. Antimicrobial resistance plasmids, typified by pRCAD0416, are characterized by the integration of an MDR region (containing genes such as *tet(X2)*, *ermD*, and *bcr*) mediated by mobile elements like IS4351. In stark contrast are the virulence-associated plasmids. Plasmid pRASD, the sole plasmid isolated from the chicken-source strain SDAU-RA1, is a prime example. Its core structure features a virulence determinant cluster containing the virulence factor *apxIB* and the toxin gene *vapD*. This cluster is located adjacent to the insertion sequence *ISRa1* and notably lacks common antimicrobial resistance genes. Notably, the close association between *ISRa1* and the *vapD* gene is recurrently observed in several other plasmids, including RA-JX, pR107, and RA961, forming a mobile *ISRa1-vapD* composite unit. This finding elucidates a key mechanism for the dissemination of virulence islands. Furthermore, some plasmids, such as pRA0726 and pRA0511, exhibit a hybrid virulence-resistance profile, simultaneously harboring the *apxIB* virulence gene alongside resistance genes like *bcr* and *cat*.

Left: Heatmap of virulence gene prevalence in *R. anatipestifer* populations from chicken, goose, and duck hosts. The color intensity corresponds to the prevalence rate for each gene in a given host group. The colored side-bar groups genes by biological function. Right: Circular genome map comparing the chicken-source strain SDAU-RA1 to six reference *R. anatipestifer* strains from chicken, goose, and duck.

(A) Genetic structural diversity and functional differentiation of the virulence plasmid pRASD. This figure shows a collinearity comparison of ten representative *R. anatipestifer* plasmids, including the novel chicken-source plasmid pRASD identified in this study. Genes are depicted as arrows, indicating their transcriptional orientation. (B) Molecular docking model of the *vapX*-like/*vapD* complex. The 3D structure of the heterodimer was predicted using AlphaFold 3, with *vapX*-like shown in yellow and *vapD* in purple. The bottom table lists the thermodynamic parameters of the protein interface; the calculated binding energy (Δ^i^G) of -8.6 kcal/mol indicates the formation of a stable complex. (C) Functional verification of the *vapX*-like-*vapD* toxin-antitoxin system. An *E. coli* BW25113 co-expression system was constructed harboring pBAD33-*vapD* (arabinose-inducible) and pKK223-3-*vapX-like* (IPTG-inducible). Bottom left: bacterial growth curves under induction with 0.2% arabinose and 1 mM IPTG. Right: spot assay results of 10-fold serially diluted cultures on induction plates. (D) Relative mRNA expression of flanking genes *apxIB* and *hin*. RT-qPCR analysis of *apxIB* and *hin* flanking the *vapX*-like-*vapD* locus in the WT, mutant, and complemented strains. The chromosomal *gyrB* gene was used as the internal control. Data represent the mean ± SD of three independent biological replicates. ns, not significant (*p* > 0.05). (E) Growth curves of the WT, mutant, and complemented strains. Data points represent three independent biological replicates. ns, not significant (*p* > 0.05). (F) Biofilm formation assay. Left: crystal violet staining in 96-well plates. Right: quantitative analysis of absorbance at OD_570_ after dissolving the crystal violet. (G) Cefazolin protection assay. (H) Oxidative stress tolerance of the WT, mutant, and complemented strains. Data represent the mean ± SD of three independent biological replicates (ns, not significant, *p* > 0.05, ****, *p* < 0.0001). (I) Persister cell assay. Statistical significance was determined using*p*-values (ns, not significant, *p* > 0.05; **, *p* < 0.01; ****, *p* < 0.0001).

### *vapX-like* and *vapD* constitute a functional toxin-antitoxin system

AlphaFold 3 structural predictions indicate that *vap*X-like and *vapD* form a stable heterodimer (Figure 7(B)), with a calculated binding free energy (Δ^i^G) of −8.6 kcal/mol, which is significantly lower than the threshold typically required for strong protein-protein interactions. Structural analysis revealed an extensive interface involving 20 residues from *vapX*-like and 19 from *vapD*, resulting in a buried surface area of 670.9 Å^2^. Within this interface, a strong interaction was observed between glutamate 32 (E32) of *vapX*-like and lysine 29 (K29) of *vapD*, while asparagine 23 (N23) of *vapD* forms a hydrogen bond network with glycine 48 (G48) and isoleucine 50 (I50) of *vapX*-like. A dual-plasmid co-expression system was established in *E. coli* to investigate the *vapX*-like/*vapD* TA system’s functionality, as confirmed by PCR analysis (Supplementary Figure 5A-C). Functional validation via spot assays demonstrated that the induction of *vapD* alone severely compromised bacterial survival across serial dilutions (10^−1^ to 10^−6^), with scant colony formation observed beyond the 10^−3^ dilution. In contrast, strains expressing *vapX*-like alone exhibited robust growth comparable to the control. Crucially, the co-expression of *vapX*-like restored colony formation to densities equivalent to the control group across all dilutions, indicating that *vapX*-like successfully rescues cells from *vapD*-induced toxicity (Figure 7C, right panel). This observation was further quantified via growth kinetics assays. Following induction, the expression of *vapD* alone caused a complete stagnation of growth over an 8-hour period, resulting in OD_600_ values significantly lower than those of the other groups (****, *p* < 0.0001). While the growth curve of the *vapX*-like-only group overlapped with that of the negative control, confirming the nontoxic nature of the antitoxin, *vapX*-like effectively neutralized *vapD* toxicity in the co-expression group, restoring both bacterial growth rate and final biomass to normal levels (significantly different from the *vapD* group; ****, *p* < 0.0001).

### Deletion of *vapX*-like-*vapD* does not affect plasmid maintenance, flanking gene expression, or growth fitness

To investigate the physiological role of the TA system in *R. anatipestifer*, we constructed a double-gene deletion mutant (Δ*vapX*-like-*vapD*) and its complemented strain (C-*vapX*-like-*vapD*) (Supplementary Figure 5D-F). To determine whether the observed phenotypes were directly regulated by the TA system rather than altered plasmid maintenance, the copy number and stability of plasmid pRASD were evaluated in the WT strain SDAU-RA1, the Δ*vapX*-like-*vapD* mutant, and the complemented strain C-*vapX*-like-*vapD*. The relative copy number of pRASD (measured via *nisB*) in the WT strain was 4.95 ± 0.19 copies per chromosome, which was not significantly different from that in the Δ*vapX*-like-*vapD* mutant (4.98 ± 0.25) or the complemented strain (4.99 ± 0.12) (Supplementary Figure 6(A)). In the plasmid stability assay, the WT, Δ*vapX*-like-*vapD*, and C-*vapX*-like-*vapD* strains all exhibited 100% plasmid retention (24/24 colonies positive for *nisB* PCR) after 30 generations of growth in antibiotic-free medium. Representative PCR results of 8 colonies per strain are shown in Supplementary Figure 6B, indicating that the deletion of the *vapX*-like-*vapD* locus did not compromise plasmid stability. Furthermore, RT-qPCR was performed to evaluate potential polar effects of the *vapX*-like-*vapD* deletion on neighboring loci. No significant differences in the transcript levels of the upstream *apxIB* and downstream *hin* genes were observed among the WT, mutant, and complemented strains (Figure 7(D)). These results demonstrate that the markerless deletion of the *vapX*-like-*vapD* locus did not disrupt the transcription of flanking genes, thereby ruling out any polar effects. Finally, to exclude the possibility that the observed defects in biofilm formation and persistence were secondary to general growth retardation, the growth kinetics of the strains in TSB were compared. The OD_600_ values of the WT, mutant, and complemented strains at each time point throughout the 24-h incubation period were virtually identical, with no significant differences in bacterial growth observed among the strains at any of the tested time points (ns; Figure 7(E)). Collectively, these findings rule out plasmid-mediated secondary effects or general fitness costs, confirming that the subsequent phenotypic defects are directly mediated by the *vapX*-like-*vapD* TA system.

### *vapX-like-vapD* mediates biofilm formation, intracellular survival, stress tolerance, and in vivo colonization

Initially, we evaluated the biofilm-forming capability of each strain using crystal violet staining. As shown in Figure 7(F), the WT strain formed a typical dense biofilm in 96-well plates, exhibiting distinct deep purple coloration upon staining. In contrast, the deletion mutant exhibited minimal staining intensity, indicating that biofilm formation was significantly impaired. Quantitative analysis further revealed that the biofilm biomass of the deletion mutant (OD_570_ ≈0.25) was significantly lower than that of the WT (OD_570_ ≈0.8; ****, *p* < 0.0001). Conversely, the biofilm-forming capability of the complemented strain was effectively restored (OD_570_ ≈0.75), showing no statistical difference from the WT (ns). To investigate the role of the *vapX*-like-*vapD* system in pathogen-host interactions, we monitored the intracellular survival dynamics of each strain within HD11 cells using a cefazolin protection assay. At the initial stage of infection (T0, 2 hours post-antibiotic treatment), intracellular bacterial loads were comparable across all groups (approximately 10^8^ CFU/mL), indicating that the TA system does not influence the efficiency of bacterial uptake by macrophages (Figure 7(G)). However, over time, the strains exhibited distinct survival patterns. Both the WT and complemented strains displayed typical intracellular persistence, maintaining viable counts of approximately 10^4^ CFU/mL at 24 hours. In contrast, the bacterial load of the deletion mutant was significantly lower than that of the control group as early as 3 hours, dropping to near the limit of detection (<10^1^ CFU/mL) by 24 hours. Statistical analysis confirmed a significant difference in survival between the deletion mutant and the WT/complemented strains at 24 hours (****, *p* < 0.0001). The complete phenotypic restoration in the complemented strain confirms that the observed defect was attributable to the absence of *vapX*-like-*vapD*.

To elucidate the physiological mechanism underlying the impaired intracellular survival of the mutant, the tolerance of the strains to oxidative stress, which represents a primary host defense mechanism within macrophages, was evaluated. Upon exposure to 2 mM H_2_O_2_ for 1 h, the Δ*vapX*-like-*vapD* mutant exhibited a significantly lower survival rate (15.50 ± 1.31%) compared to the WT strain SDAU-RA1 (45.22 ± 2.62%) (Figure 7(H)). The complemented strain C-*vapX*-like-*vapD* fully restored the oxidative tolerance phenotype (41.81 ± 2.39%, ns compared to WT). These results indicate that the *vapX*-like-*vapD* system contributes to intracellular persistence by protecting *R. anatipestifer* against host-derived oxidative stress.

The role of the *vapX*-like-*vapD* system in the bacterial response to a lethal antibiotic stress was investigated by determining time-kill kinetics on log-phase cultures treated with cefazolin (Figure 7(I)). As antibiotic exposure time increased, survival profiles differed significantly according to the results. Both WT and complemented strains showed significant tolerance to antibiotics, slow declining counts and biphasic kinetics of killing indicative of persister formation. Conversely, the deletion mutant showed a strong susceptibility to cefazolin, with declining cell counts. At 2 and 6 hours post-treatment, significant differences in bacterial loads between the deletion mutant and complemented strain were observed (**, *p* < 0.01 and ****, *p* < 0.0001, respectively). After 24 hours, the viable counts for the deletion mutant had fallen to approximately 10^1.7^ CFU/mL, a decrease of nearly 4 orders of magnitude from the initial value, while the WT and complemented strains remained at approximately 10^5^ CFU/mL. The survival curve of the complemented strain was similar to the WT (ns), indicating a full restoration of the tolerance phenotype. Finally, systemic colonization was evaluated in chickens at 48 hpi (Supplementary Figure 6C). The Δ*vapX*-like-*vapD* mutant showed significantly lower bacterial loads in the liver (3.92 Log_10_ CFU/mL), spleen (4.15 Log_10_ CFU/mL), and blood (2.38 Log_10_ CFU/mL) compared to the WT strain (5.82, 6.12, and 4.48 Log_10_ CFU/mL, respectively; ***, *p* < 0.001, ****, *p* < 0.0001). These colonization defects were fully restored in the complemented strain. These results indicate that the *vapX*-like-*vapD* system is crucial for the systemic colonization of *R. anatipestifer* in chickens.

### Pan-genome structure of *R. anatipestifer* from different hosts

**Figure 8.**
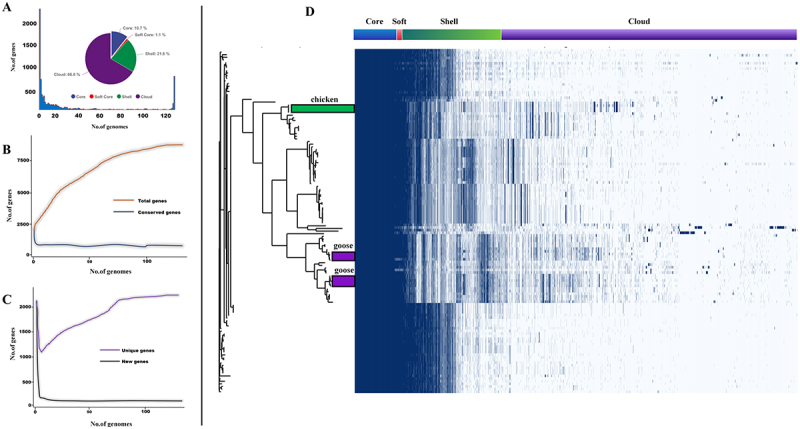
Pangenome structure of *R. anatipestifer* from different hosts.

To elucidate the population genomic structure and diversity of *R. anatipestifer*, we conducted a pan-genome analysis. The results revealed a quintessential open pan-genome: its core (944; 10.7%) and soft-core (95; 1.1%) genomes together comprised only 11.8% of the total gene families, whereas the accessory genome, composed of shell (1,910; 21.6%) and cloud (5,876; 66.6%) genes, accounted for a substantial 88.2% (Figure 8(A)). The “U-shaped” distribution of gene frequencies visually illustrates the coexistence of a conserved core genome and a vast number of rare genes, indicating the species’ high genetic diversity and strong adaptive potential (Figure 8(A)). The pan-genome size steadily increased with the addition of each strain and did not reach a plateau after the inclusion of all 130 strains, totaling over 8,800 gene families; in contrast, the core genome stabilized at approximately 1,000 gene families (Figure 8(B)). The open nature of the pan-genome was further confirmed by the dynamic analysis of new and unique genes (Figure 8(C)): dozens of new genes were identified with the inclusion of each subsequent genome, while the cumulative number of unique genes exceeded 2,200. Furthermore, a gene presence/absence matrix revealed that the distribution patterns of accessory genes correlated closely with the strains’ phylogenetic relationships, indicating that gene gain and loss are key drivers of population differentiation (Figure 8(D)).

(A) Pangenome composition of 130*R. anatipestifer* genomes. The pie chart illustrates the classification of genes into core, shell, and cloud categories. The histogram displays the frequency distribution of genes across the genomes. (B) Pangenome and core genome accumulation curves. The plot shows the number of total gene families (pangenome) and core gene families as a function of the number of genomes added. (C) New and unique gene accumulation curves. The plot shows the number of new genes discovered with the addition of each genome and the cumulative number of unique genes. (D) Heatmap of the gene presence/absence matrix. Each row corresponds to a strain, ordered according to the phylogenetic tree, and each column represents a gene family. Dark blue indicates gene presence, while white indicates absence.

A comparative analysis of the accessory genomes (Supplementary Table 10) revealed population genetic differences. We found that chicken-source isolates were enriched in genes associated with virulence regulation and stress resistance. For instance, *gspA* (a type II secretion system component) and *ptk* (a virulence regulatory kinase) may directly enhance pathogenicity, while *nlpE* (an envelope stress protein) and *ruvA* (a DNA repair protein) likely contribute to resisting host immune pressure. In contrast, goose-source isolates specifically harbored a different set of genes related to nutrient acquisition and metabolic adaptation. Among these, *entS* (a siderophore transporter) is crucial for competing for essential iron within the host. Additionally, the presence of genes such as *linA*, *yhcG*, and *smc* suggests unique physiological adaptations to the goose host environment.

## Discussion

*R. anatipestifer* is the primary etiological agent of infectious serositis in waterfowl, posing a significant threat to the global waterfowl industry [24]. Traditionally, its host range was considered restricted to waterfowl such as ducks and geese. However, the detection rate in chicken populations has recently increased significantly, suggesting an expansion of its host range [3,4]. This trend poses a severe biosecurity challenge to the much larger poultry industry. Nevertheless, the molecular mechanisms driving the cross-host transmission of this pathogen remain poorly understood, and systematic comparative genomic studies of strains from different hosts are lacking. In this study, we isolated and characterized a highly virulent chicken-source strain and, for the first time, conducted a systematic comparative genomic analysis to elucidate the genetic differentiation, host adaptation, and the molecular basis of cross-species transmission among strains from these different hosts.

Our core-genome SNP phylogenetic analysis revealed a unique population structure characterized as “globally mixed, yet locally clustered.” Strains from the primary host, ducks, form the genetic backbone of the population, while chicken- and goose-source strains are interspersed throughout. This pattern provides strong evidence for continuous gene flow between host species, rather than long-term, isolated evolution. This corroborates findings for other important zoonotic pathogens, such as *Campylobacter jejuni*, whose population structure has also been shown to be shaped by host-jumping events [25,26]. Notably, we observed significant local clustering. For instance, chicken-source strains were predominantly ST134, while goose-source strains mainly belonged to ST4 and ST64, and these clusters were also highly concentrated geographically in Jiangsu Province, China. This highlights the critical role of geographical proximity and mixed-farming practices in the cross-species transmission and local outbreaks of the pathogen. Once a strain successfully “jumps” to a new host, the shared geo-ecological niche and farming environment exert uniform selective pressure, promoting the rapid proliferation and dissemination of specific clonal groups, such as ST134 in chickens, thereby forming the observed clustering patterns. This conclusion is consistent with findings for Avian Pathogenic *E. coli* (APEC) and Extraintestinal Pathogenic *E. coli* (ExPEC), where the geo-ecological niche has also been identified as a key driving force in shaping population structure and host adaptation [27–29]. Furthermore, ASR provided robust evolutionary evidence that ducks act as the primary reservoir of *R. anatipestifer*, driving not only multiple independent spillover events to chickens but also frequent host transitions between ducks and geese.

This study characterizes the evolution of the *R. anatipestifer* resistome. A prominent finding was a dramatic increase in the prevalence of several key ARGs between 2021 and 2025: the florfenicol resistance gene *floR*, the β-lactamase gene *bla*_*OXA-209*_, and *tet(X)*, which confers resistance to the last-resort antibiotic tigecycline. This trend parallels the evolutionary patterns of resistance observed in swine- and poultry-derived *E. coli* in China, where selective pressures from clinical and agricultural antibiotic use are known to directly shape the pathogen’s resistome [10,12,30]. In the chicken-source strain SDAU-RA1, the *tet(X)* gene was not found in isolation; instead, it was integrated into a large and highly complex composite genomic island flanked by phage elements. This GI incorporates at least ten distinct ARGs, including *tet(X)*, *bla*_*OXA-209*_, *ErmF*, and *DfrA*, with a scale and complexity far surpassing anything observed in the waterfowl-derived strains. This architecture, which consolidates multiple ARGs onto a single mobile genetic unit, represents a stark and concerning manifestation of virulence-resistance co-linearity. The formation mechanism bears a strong resemblance to the resistance islands identified in critical human pathogens such as *Acinetobacter baumannii* and hypervirulent *Klebsiella pneumoniae* (hvKP) [31–33]. This process involves the synergistic interplay of genomic islands, integrons, and phages to capture and assemble ARGs from diverse origins into specific genomic hotspots. Crucially, we identified the *parD1/parE1* genes, which are associated with plasmid partitioning systems, located upstream of this GI [34]. This finding is a strong indicator that the composite resistance island possesses the potential for efficient, plasmid-mediated horizontal gene transfer.

The adaptation of a pathogen to a new host typically involves the acquisition or modification of specific virulence factors [35]. Our analysis revealed that chicken-source *R. anatipestifer* strains, as a population, are significantly enriched in a suite of virulence genes that may facilitate adaptation to the avian host. These include *TssL*, encoding a core protein of the Type VI Secretion System; *wbjD/wecB*, key enzymes for capsular polysaccharide synthesis; and *pglC*, involved in the glycosylation of virulence proteins. This may represent a primary evolutionary trajectory for host adaptation, a hypothesis that warrants further investigation. Intriguingly, the pathogenic strain SDAU-RA1, characterized in this study, completely lacks these three genes, which underscores the complexity and diversity of the pathogenic mechanisms of *R. anatipestifer*. The pathogenicity of SDAU-RA1 evidently relies on an alternative virulence toolkit. The plasmid pRASD, identified for the first time in a chicken-source strain in this study, offers a compelling explanation. This plasmid functions as a highly specialized virulence plasmid, whose core functional module is a virulence island containing the virulence factor *apxIB* and the *vapD* toxin gene, yet it carries no common antimicrobial resistance genes. This modular partitioning of virulence and resistance functions onto different genetic elements confers remarkable adaptive flexibility, allowing the bacterium to acquire traits on demand in response to specific environmental pressures. Analogous to the well-characterized IUC3-positive virulence plasmids responsible for the hypervirulence and transmissibility of hvKP [7,36], we further discovered that this virulence island is tightly linked with the insertion sequence *ISRa1*. This association forms a mobile composite unit, designated *ISRa1-vapD*, which provides a clear molecular basis for the modular dissemination of virulence genes. Crucially, by integrating AlphaFold 3 structural prediction with biochemical assays we demonstrate that *vapX*-like-*vapD* operates as a bona fide type II TA system. Even though there is low sequence homology between the *vapX*-like gene and classical *vapX*, structural modeling indicated that *vapX*-like forms a high-affinity heterodimer (Δ^i^G = −8.6 kcal/mol) with *vapD* through E32-K29 salt bridge and a hydrogen bond network, thereby effectively neutralizing the toxin. This finding coincides with recent information concerning the diversity of *vapD* regulators [37], such as the newly identified antitoxin families *vapY* and *vapW*. *VapY* has no sequence similarity to *vapX* and *vapW* has evolved as a catalytically dead toxin homolog that prevents *vapD* oligomerization. These findings suggest that the *vapX*-like-*vapD* system potentially acts as a regulatory switch for *R. anatipestifer* in response to environmental stress. The *vapD* toxin typically contains a PIN domain with predicted ribonuclease activity. Under stress conditions, its activation is hypothesized to cleave cellular RNAs, thereby potentially leading to translation inhibition and metabolic arrest [38]. This transition into a transient dormant state may promote the formation of biofilms and persister cells. Consequently, deletion of this system appears to impair biofilm formation and compromise the bacterium’s capacity to maintain a persistent state under antibiotic pressure or within macrophages, potentially accelerating clearance by the host immune system. Ultimately, this proposed “biofilm-persister” dual defense mechanism likely facilitates bacterial colonization in harsh external environments and may serve as a key strategy for establishing persistent infections and evading antibiotic clearance in host tissues [39].

R. *anatipestifer* exhibits a typical open pangenome structure, with its accessory genome accounting for a remarkable 88.2% while the core genome constitutes only 10.7%. This U-shaped distribution and open pangenome curve indicate immense genetic plasticity, which provides the genetic foundation for its adaptation to diverse environments and new hosts [35,40–42], a common characteristic of pathogens like *E. coli*. Comparative analysis of accessory genomes from different host-derived strains revealed distinct signatures of host adaptation. Chicken-source strains were significantly enriched in genes related to virulence regulation (*ptk*) [43,44], the Type II Secretion System (*gspA*) [45], and stress resistance (*nlpE*, *ruvA*) [46,47], suggesting they may have evolved enhanced capabilities to resist host immune clearance and repair DNA damage. In contrast, goose-source strains were specifically enriched in nutrient acquisition genes, such as the siderophore transporter gene *entS*, reflecting a highly efficient iron acquisition strategy tailored to the goose host microenvironment [48]. These differences clearly delineate the divergent survival strategies employed by the bacterium to adapt to different hosts.

Lastly, a key limitation of this study is the host-associated sample size imbalance, as the number of available chicken-derived *R. anatipestifer* genomes is substantially smaller than those from ducks and geese. This disparity, which stems from the scarcity of sequenced chicken-derived isolates in public databases, may constrain the statistical power of our genomic enrichment analyses. Consequently, future studies incorporating larger and more balanced cohorts of sequenced chicken genomes are required to validate these findings.

In summary, this study systematically elucidated the patterns of cross-host transmission, evolution, and adaptation of *R. anatipestifer*. We conclude that its cross-host transmission is not a deliberate evolutionary event but rather a multi-stage, opportunistic process facilitated by vulnerabilities within modern intensive farming systems. Personnel, vehicles, equipment, and contaminated water sources serve as key vectors for introducing this waterfowl pathogen to terrestrial poultry (chickens). Concurrently, prevalent respiratory and immunosuppressive viral infections compromise flock barriers and weaken immunity, creating a window of opportunity for *R. anatipestifer* to emerge as a lethal pathogen [49,50]. Once established within a chicken flock, its open pangenome facilitates the synergistic evolution of virulence and antimicrobial resistance. While geographic proximity promotes transmission, intense antibiotic selection pressure accelerates the selection of “superbugs” equipped with composite resistance islands. Notably, the discovery of plasmid pRASD and its *vapX*-like-*vapD* system offers a new perspective on how bacteria utilize plasmid-encoded TA systems to coordinate environmental adaptability with virulence expression. Although the bacterium has acquired the capacity to infect chickens, it has not evolved strict host specificity. Ongoing gene flow indicates that it remains a shared pathogen reservoir circulating between waterfowl and terrestrial poultry. These findings not only deepen our understanding of the mechanisms of pathogen cross-species transmission but also serve as a critical warning regarding the deteriorating biosecurity landscape in the poultry industry.

## Supplementary Material

Supplementary Figure 6.tif

Supplementary Figure 1.jpg

Supplementary Figure 5.tif

Supplementary Materials.docx

Supplementary Figure 3.tif

Supplementary Figure 2.jpg

## Data statement

All data and results from this study are openly accessible. The raw data supporting the research findings, as well as all supplementary materials, have been deposited in the Figshare repository (https://doi.org/10.6084/m9.figshare.30184042). The complete genome sequence of SDAU-RA1 has been submitted to NCBI (https://www.ncbi.nlm.nih.gov/nuccore/CM126355.1/) under the accession number CM126355.1 (chromosome).
