# Supplementary figures and images for: Cross-host transmission of Riemerella anatipestifer to chickens: Genomic evolution and identification of the novel vapX-like-vapD toxin-antitoxin system
Source: Virulence. 2026 Jul 30;17(1):2711521. doi: 10.1080/21505594.2026.2711521 (PMC13432907; doi:10.1080/21505594.2026.2711521)

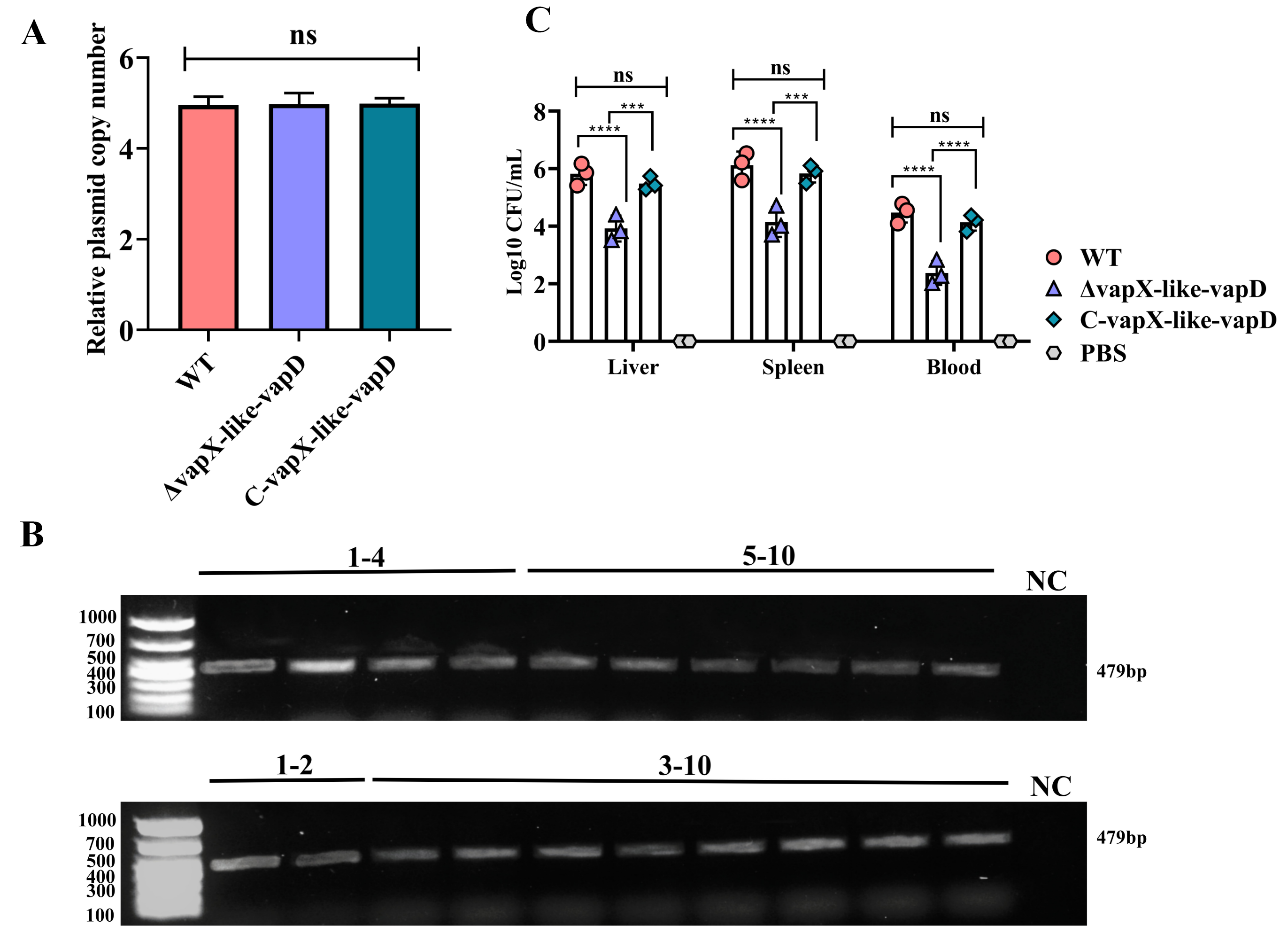

Supplement: Supplementary Figure 6.tif [file KVIR_A_2711521_SM9828.tif]

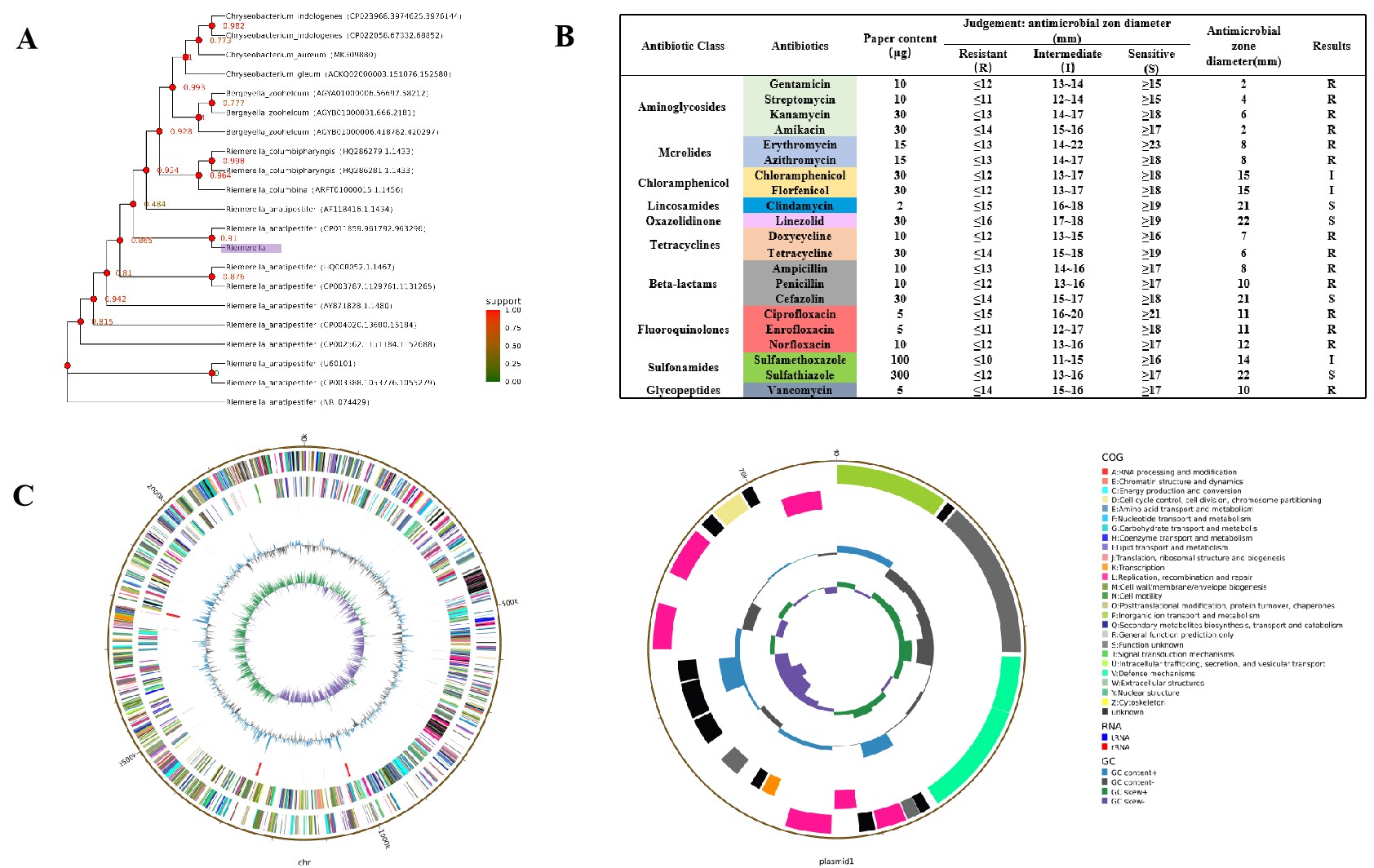

Supplement: Supplementary Figure 1.jpg [file KVIR_A_2711521_SM9827.jpg]

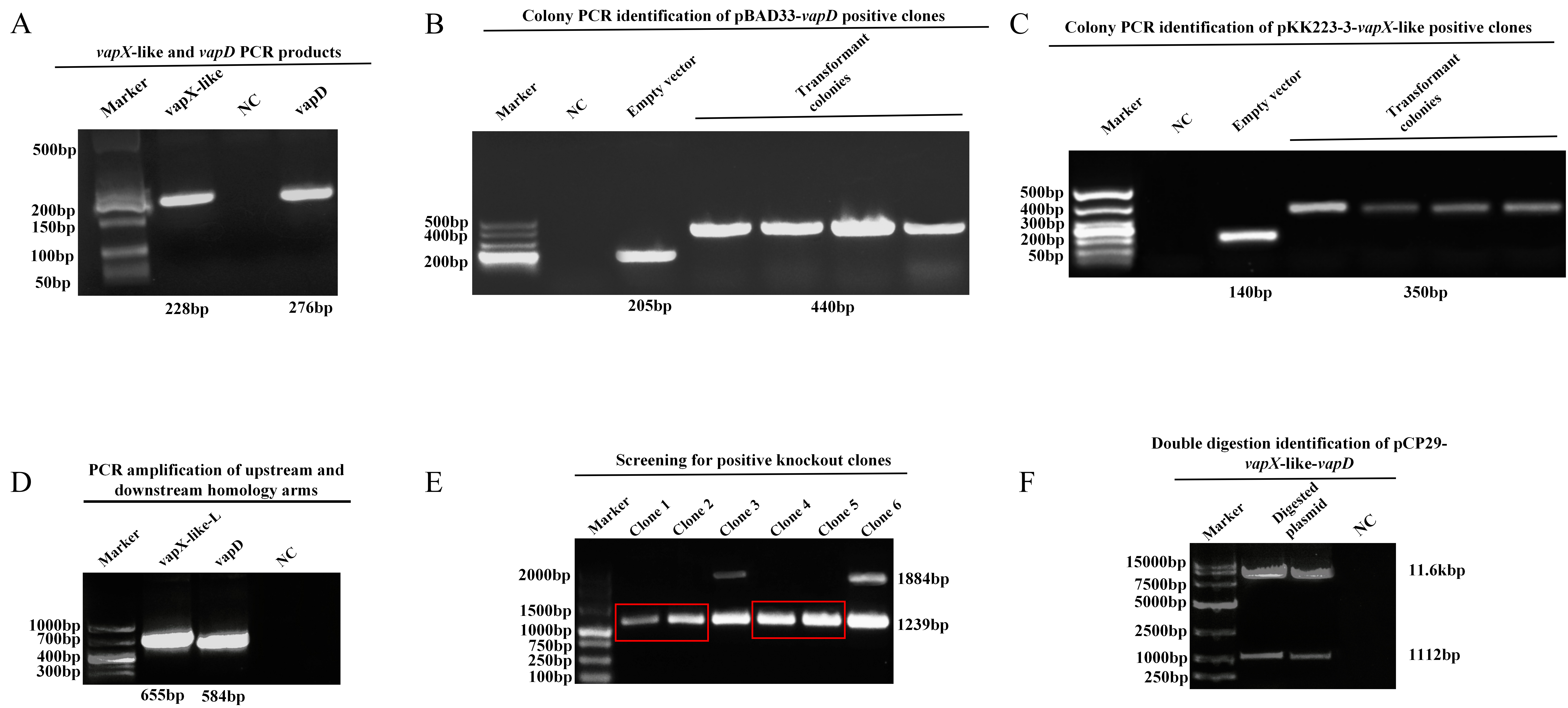

Supplement: Supplementary Figure 5.tif [file KVIR_A_2711521_SM9826.tif]

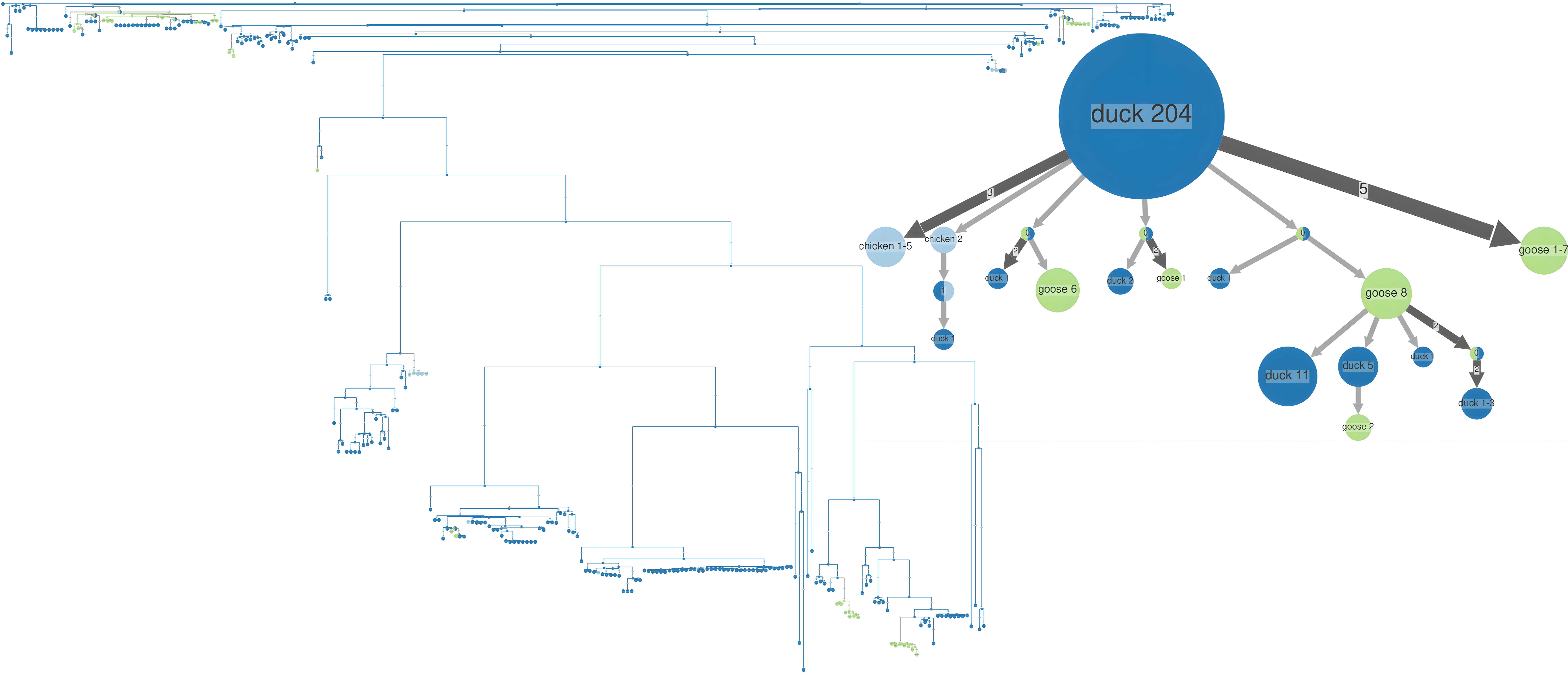

Supplement: Supplementary Figure 3.tif [file KVIR_A_2711521_SM9824.tif]

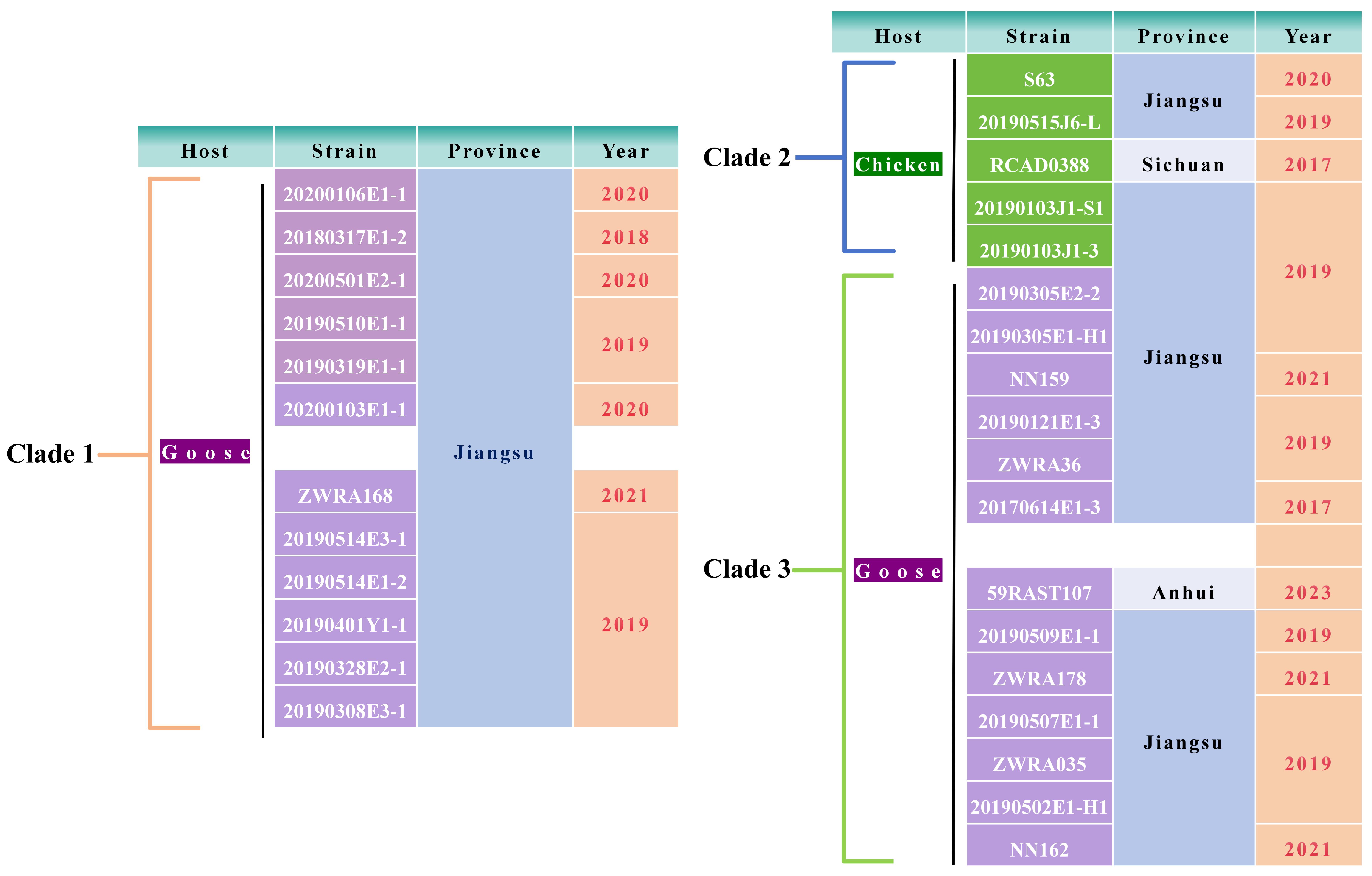

Supplement: Supplementary Figure 2.jpg [file KVIR_A_2711521_SM9823.jpg]
