# Supplementary material for: Cross-host transmission of Riemerella anatipestifer to chickens: Genomic evolution and identification of the novel vapX-like-vapD toxin-antitoxin system
Source: Virulence. 2026 Jul 30;17(1):2711521. doi: 10.1080/21505594.2026.2711521 (PMC13432907; doi:10.1080/21505594.2026.2711521)
Supplement: Supplementary Materials.docx [file KVIR_A_2711521_SM9825.docx]

**Supplementary Materials**

**Supplementary Figure 1** Biological characteristics of SDAU-RA1.

(A) Phylogenetic analysis based on 16S rRNA gene sequences. The neighbor-joining tree was constructed using MEGA X, and the statistical reliability of the branches was assessed by 1000 bootstrap replicates. (B) Antimicrobial susceptibility profile of SDAU-RA1. (C) Circular map of the SDAU-RA1 chromosome (left) and plasmid (right).

**Supplementary Figure 2** Analysis of geographical origins revealed a distinct clustering pattern.

To investigate this, we specifically examined the source attributes of the clustered chicken- and goose-source isolates from the SNP phylogenetic tree. This revealed that the goose-derived isolates in Clades 1 and 3, along with the chicken-derived isolates in Clade 3, predominantly originated from Jiangsu Province, China.

**Supplementary Figure 3** ASR of host transition events for *R. anatipestifer*.

The maximum-likelihood phylogenetic tree (left) and the reconstructed host transmission network (right) were generated using PastML. Colors represent different host species: blue for ducks, green for geese, and light blue for chickens. In the transmission network, circles (nodes) represent ancestral or sampled host states, with the circle size and internal numbers indicating the number of isolates in each cluster. Arrows indicate the direction of host transition (transmission) events, and the numbers on the arrows (weights) represent the frequency of independent transition events.

**Supplementary Figure 4** Heatmap of ARGs and VFs across *R. anatipestifer* isolates.

The dendrogram on the left clusters isolates based on their gene profiles. Blue and purple cells represent the presence of ARGs and VFs, respectively.

**Supplementary Figure 5** Agarose gel electrophoresis of PCR products for *VapX*-like/*VapD* system identification.

1. *vapX*-like and *vapD* PCR products.(B) Colony PCR identification of pBAD33-*vapD* positive clones. (C) Colony PCR identification of pKK223-3-*vapX*-like positive clones. (D) PCR amplification of upstream and downstream homology arms. (E) Screening for positive knockout clones (Δ*vapX*-like-*vapD*). (F) Double digestion identification of pCP29-*vapX*-like-*vapD.*

**Supplementary Figure 6** Evaluation of pRASD plasmid maintenance and in vivo colonization.

(A) Relative copy number of plasmid pRASD in the WT, mutant, and complemented strains. (B) PCR verification of plasmid stability after 30 generations of passage. Top gel: Lanes 1–4 (WT colonies), Lanes 5–10 (Δ*vapX*-like-*vapD* mutant colonies), NC (negative control). Bottom gel: Lanes 1–2 (ΔvapX-like-*vapD* mutant colonies), Lanes 3–10 (C-*vapX*-like-*vapD* complemented colonies), NC (negative control). (C) Bacterial loads (Log_10_ CFU/mL) in the liver, spleen, and blood of 7-day-old chickens at 48 hpi. PBS was used as the negative control. Data represent the mean ± SD. ns, not significant (*p* > 0.05); ***, *p* < 0.001; ****, *p* < 0.0001.

**Supplementary Table 1** Source Information for the 283 *R.anatipestifer* Isolates.

**Supplementary Table 2** Predicted ARGs and VFs in the 280 *R. anatipestifer* isolates. The values represent the number of identified genes.

**Supplementary Table 3** Prevalence of ARGs in 280 isolates from different hosts (chicken, duck, and goose), defined as the percentage of isolates harboring each gene.

**Supplementary Table 4** Prevalence of ARGs in 280 *R. anatipestifer* isolates across four time periods.

**Supplementary Table 5** Prevalence of ARGs in 280 *R. anatipestifer* isolates from different geographical regions.

**Supplementary Table 6** Prevalence of VFs in 280 *R. anatipestifer* isolates from different hosts.

**Supplementary Table 7** Accession Numbers of Plasmid Genes for Synteny Analysis.

**Supplementary Table 8:** Primer sequences and sizes of PCR fragments.

**Supplementary Table 9** STs of the 280 *R. anatipestifer.*

**Supplementary Table 10** Presence-absence matrix of pangenome genes from 130 *R. anatipestifer* strains.
